# Exploring the Volume Paradox: Comparing Different Formulas for Estimating the Wood Volume of Trees and Logs in Nepal

**DOI:** 10.1155/sci5/4884890

**Published:** 2026-01-21

**Authors:** Pawan Karki, Shambhu Dangal, Edwin Cedamon

**Affiliations:** ^1^ Nepal Country Office, Regional Community Forestry Training Center for Asia and the Pacific (RECOFTC), Lalitpur, Nepal; ^2^ School of Agriculture, Food and Wine, The University of Adelaide, Adelaide, South Australia, Australia, adelaide.edu.au

**Keywords:** harvesting loss, Huber’s formula, Quarter girth formula, timber volume

## Abstract

Accurate estimation of tree and log volume plays a pivotal role in a wide range of applications within the field of forestry, especially in biomass assessment and forest management. The effectiveness and applicability of formulas for volume estimation have recently become a topic of extensive deliberation among forestry officials and a diverse array of stakeholders. This study conducts a comparative analysis between the Huber and allometric equation with the Quarter girth formula and a formula based on form factor to estimate the volume of standing trees and logs. Field measurements of 168 felled trees in coupes and randomly selected 1192 logs were examined in this study. The findings of the study revealed a decrease of approximately 0.8% in timber volume and a substantial increase of 78.6% in firewood volume while adopting the allometric equation when compared to the formula based on form factor. Similarly, the application of Huber’s formula resulted in a 27.34% increase in volume compared to the Quarter girth formula. ANOVA demonstrated highly significant differences in the average volume per tree among the four formulas (*p* value = 0.00004) for both standing tree and log volume, and further analysis using Tukey’s HSD indicated that the Quarter girth and form factor–based formula for standing trees differed significantly from the allometric equation for standing timber, while other formula combinations did not show significant differences. Timber losses in the felling and bucking stage with the Quarter girth and Huber formula were 37.06% and 19.86%, respectively. Moreover, paired *t*‐test at 5% level of significance revealed that there was a significant loss in both tree felling using both formulas.

## 1. Introduction

Forests are renewable natural resources that supply a variety of commodities and services to meet social and environmental demands [1]. To ensure the availability of these resources, efficient forest production and balanced usage are essential. Nepal is pioneer in decentralized forest management in the Global South, having practiced community‐based forest management (CBFM) for more than 4 decades [2, 3]. The National Forestry Plan of 1976 and the Forest Sector Master Plan of 1988 set the foundation for the CBFM [4]. The Forest Act 1993, the Forest Regulation 1995, the Forest Act 2019, and the most recent Forest Regulation 2022 all promoted this process. However, efficient forest usage faces various obstacles, mostly due to resource trade‐offs [5] and continued forest harvesting policies and practices [6].

Nepal’s community forests are critical resources that are managed collectively by local communities for their livelihoods and environmental conservation [7]. Timber has always been one of the key income sources in CBFM in Nepal [8, 9], making it a major source of contestation in the mid‐hilly region of Nepal. Besides grade, which affects the price per unit, it is necessary to precisely estimate volume and weight to quantify the amount of merchantable timber. So, estimation of timber quantity affects the economic vitality of the timber business and the community’s benefits. Besides the effects of the accuracy of the estimates on revenue, they also have a function on a societal level, serving as input into records and being used in industrial statistics. However, the forest area is lower than the average global per capita forest land available in Nepal [10–12]. Within this forest area, the average number of stems greater than 10 cm in diameter is 430 ha^−1^ [12]. Moreover, despite increasing efforts of the government and nongovernment actors, the mean stem volume per unit area of Nepal was decreased [11, 12].

The accurate estimate of tree and log volumes is essential for effective planning, forest inventory, and resource allocation. Before the Forest Regulation 2022 and its subsequent amendment, the Quarter girth formula is commonly utilized for saw log volume estimation, along with form factor–based formulas for standing trees. The Quarter girth formula, which calculates the volume of a log based on its circumference, was preferred for its simplicity and ease of use in the field. However, form factor–based formulas consider the shape and taper of standing trees, providing easier estimates of their volumes. It has been observed that the Quarter girth formula typically yields 78.5% of the actual cubic volume of the log, allowing for a 21.5% allowance for lumber processing requirements, including slabs, edgings, and sawdust [13].

Forest Regulation (2022) introduced a new provision that affected the volume estimation method of both standing trees and felled trees. Initially, the regulation mandated the use of specific formulas, such as Huber and allometric equations developed by Sharma and Pukkala [14] for volume estimation of logs and trees. However, the amendment of Forest Regulation (2022) introduced significant changes, replacing the Huber formula with again Quarter girth formula. These changes caused misunderstandings and sparked debates among forest professionals and relevant stakeholders. So, the objective of this study was to compare different methods for estimating the volume of standing trees and logs used in Nepal. Specifically, it examines the Huber and allometric equations, with the Quarter girth formula, and a formula based on form factor. By conducting a comparative analysis of these formulas, the study aims to assess their accuracy and effectiveness in estimating tree volume, providing valuable insights to forest practitioners. In addition, this study served as a baseline that fills the knowledge gap on timber volumes by different formulas and timber loss along the timber production chain and suggests corrective actions to adopt for its control.

## 2. Materials and Methods

### 2.1. Study Area

**Figure 1:**
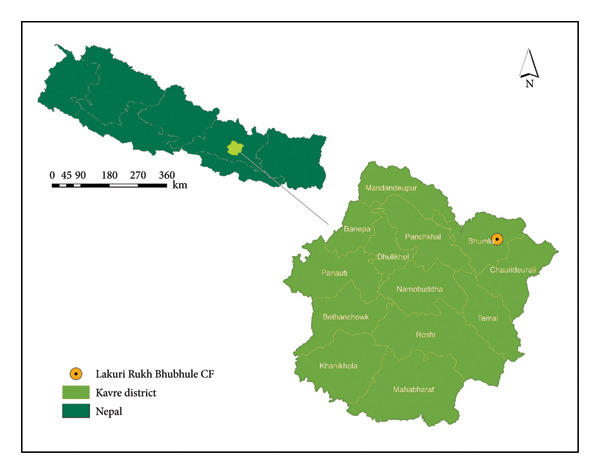
Location map of study area.

The assessment was conducted in Lakuri Rukh Bhubhule Community Forest of Bhumlu Rural Municipality of Kavrepalanchok District in Nepal, which was handed over to forest user groups comprising 88 HH in 1995 (Figure 1). The total forest area of 39.2 ha is dominated by matured planted pine (*Pinus patula*) managed through a series of thinning and regeneration felling to promote natural regeneration (Table 1).

**Table 1:** Description of the study site.

CFUG name	Handover date as CF	Total forest area (ha)	Total HHs involved	Forest type	Major forest species
Lakuri Rukh Bhubhule Community Forest, Kavre, Nepal	1995	39.2	88	Plantation pine forest	*Pinus patula, Pinus wallichiana, Alnus nepalensis, and Schima wallichiana*

At this assessment site, over 80% of the forest is dominated by Patula pine (*Pinus patula*) species with good market value. The sale of pine timber contributes around 80% of the total annual income of this CFUG. The CFUG follows the forest operational plan that involves thinning, regeneration felling, and other activities aiming to promote regeneration.

### 2.2. Data Collection

A total of 168 pine trees were harvested in the felling coupe of CF during February and March 2023. The diameter at breast height (DBH) at a height of 1.3 m was measured for the standing trees. The total height (H) and the height of the tree bole at the first circular branching of the stem were also recorded. The DBH and total height were used to calculate the standing volume of each individual tree [15, 16]. Detailed information and identification of the sampled trees after felling were recorded, including geographic coordinates, tree number, crown radii in four cardinal directions, and damage types. Following the felling, the actual height and bole height of the trees were measured. The felled trees were then categorized into desirable log sizes (6 ft). The tree fellers performed the log conversion as part of their routine work. The length and over‐bark diameters at the middle positions of each log were measured. The Apresys portable laser Rangefinder 5–550 M Pro was used for estimating the standing tree total height and tree bole height. The diameter tape was employed to measure the DBH and different stem diameters of the logs. Linear tape was used to measure the felled tree’s actual height/length, bole height, and log length.

### 2.3. Data Analysis

All data from field measurements were analyzed using Statistical Package for the Social Sciences (SPSS) to rigorously assess and compare the estimated volumes of logs and trees. To achieve this, we employed a combination of statistical techniques, including a paired *t*‐test to determine whether there were significant differences in volume between logs and trees, analysis of variance (ANOVA) to explore potential variations among different types or categories of logs and trees, and the Tukey HSD test to pinpoint specific pairings that exhibited significant volume disparities. This comprehensive analysis aimed to provide valuable insights into the volume disparities of logs and trees, facilitating informed decision‐making in the realm of forestry and resource management in Nepal.

### 2.4. Comparison: Estimated Standing Volume, Felled Volume, and Log Volume

The volume estimation of a standing tree was performed using an allometric equation and a conventional volume calculation formula based on the form factor. This requires the measurement of the DBH and the total height of the tree. The coefficients specified for pine species, in accordance with Forest Regulation 2022, were adopted for the parameters a, b, and c [14]. Furthermore, a uniform form factor of 0.50, recommended by the Department of the Forest, Government of Nepal, was applied to all species across the country [17]. For determining the volume of a felled tree, the same equation was employed using the measured attributes of DBH and total height. To calculate the volume of the logs, the Quarter girth formula and Huber formula, as prescribed by both the old and new forest regulations, were utilized. To investigate potentially significant differences in tree and log volume resulting from the utilization of these two formulas, a paired *t*‐test was conducted. The formulas used for estimating standing tree and log volumes are presented in Table 2.

**Table 2:** Formulas used for calculating different volumes.

Estimates	Types	Model used	Description of notation
Volume of standing tree	Form factor–based formula	*V* * = π* *d* ^2^ ×* h/*4 × f.f	*V* = volume of standing tree in m^3^, *d* = DBH in cm, *h* = tree height, and f.f = form factor
	Allometric equation [14]	Ln (*V*) = *a* + *b*Ln (*d*) + *c*Ln (*h*)	Ln = natural log base; *V* = volume in m^3^; *d* = DBH in cm; *h* = total tree height in m; and a, b, and c are coefficients of species.

Log volume	Quarter girth formula	*V* = (g/4) 2 × L	*V* = log volume in m3, *g* = girth, and *L* = length of the log in m,
	Huber’s formula	*V* = Sm × L	*V* = log volume in m^3^, Sm = middle basal area in m^2^, *L* = length of the log in m, and basal area (*S*) = πd2/4 in m^2^

## 3. Results

### 3.1. Overview of Tree and Log Dimensions and Estimated Volume

Table 3 below presents a basic statistical description of a subset of data, i.e., tree DBH and middle diameter of logs. The total estimated volume is the total timber volume of the sampled trees and logs using the allometric equation of Sharma and Pukkala [14] and the volume calculation formula based on the form factor.

**Table 3:** Statistical parameters and estimated volume of sampled trees and logs.

Type	No.	DBH/dm (cm)^∗^					Height/length (m)^∗∗^					Total estimated volume (m^3^)	
		Mean	Min	Max	S.D. (*σ*)	S.E.	Mean	Min	Max	S.D. (*σ*)	S.E.	Previously used	New
Tree	168	34.01	14.00	70.80	11.67	0.90	17.83	7.00	32.00	3.86	0.30	5868.49	7401.66
Log	1192	26.44	10.52	68.76	9.92	0.29	7.63	4.00	8.00	0.78	0.02	4658.58	5931.47

^∗^DBH = diameter at breast height of a standing tree in cm measured over bark taken at the height of 1.3 m from the ground, and dm is over‐bark mid‐diameter of logs.

^∗∗^Height/length = total height of the standing tree and length of log in m.

### 3.2. Sizes of Sample Trees

**Figure 2:**
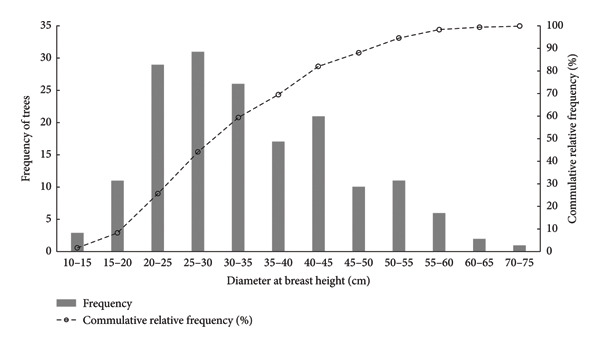
Stock table of sample trees from Lakuri CF harvesting coupe.

The sample trees consisted of harvested trees from Lakuri CF with DBH ranging from 14 cm to 71 cm. It is notable in Figure 2 that the distribution of sample trees to DBH classes followed somehow a normal distribution. While the bars in Figure 2 show that the highest frequencies were in the DBH classes 20–30 cm, which are considered medium‐sized trees, that represents about one‐third of the sample trees. Just over half of the sample were large trees comprising DBH of 30 cm–71 cm (see dotted line representing cumulative relative frequency). The wide range of tree sizes included in the sample means that the volume formulas tested across DBH sizes increased the confidence on results of the analysis. Moreover, the wide array of sample trees allowed investigation of the influence of tree sizes on the differences in volume estimates across the four volume formulas for log and standing trees.

### 3.3. Volume Comparison of Standing Trees

**Figure 3:**
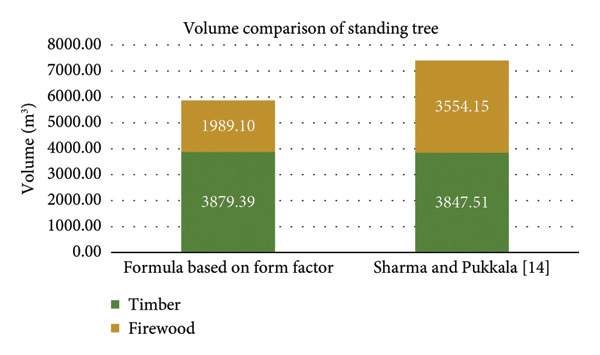
Comparative chart of tree volume using previously used formula based on form factor and allometric equation.

The allometric equation estimates a timber volume slightly lower than the form factor–based formula, implying a minor decrease of approximately 0.8% compared to the form factor–based method (Figure 3). Further, the difference between the two methods was noticed to be relatively small. But, for firewood, the previously used formula estimated volume of 1989.10 m^3^, whereas the allometric equation estimated to be significantly higher, i.e., 3554.15 m^3^, indicating an increase of approximately 78.6% (1565.05 m^3^) when using the new method for firewood calculation.

In general, the previously used formula for tree volume calculation based on form factors is commonly used for estimating tree volume. These methods involve calculating the form factor, which is a conversion factor that accounts for the shape and taper of tree stems. While there are various form factor–based equations available for different tree species, they often require detailed measurements and assumptions about stem shape, which can introduce uncertainties and potential inaccuracies. While volume calculation of trees using allometric equations offers several benefits compared to previously used methods based on form factors. Allometric equations are based on statistical models that consider various tree parameters, such as diameter, height, and other relevant variables to estimate volume. Unlike form factor–based methods, allometric equations provide a more flexible and adaptable approach as they can be developed specifically for different tree species or local conditions. Previously used form factor–based formulas often focus solely on the stem volume, neglecting the contribution of branches, which can be significant in certain tree species or growth forms. By incorporating branch volume, allometric equations capture a more complete representation of the tree’s overall volume which increases the volume of firewood. This improved accuracy is crucial for various applications, including forest inventory, timber management, and carbon sequestration assessments. This allows for improved accuracy and reduced bias in volume estimation. Overall, allometric equations offer a more precise, versatile, and user‐friendly alternative to previously used form factor–based methods for tree volume calculation [18].

### 3.4. Volume Comparison of Log

**Figure 4:**
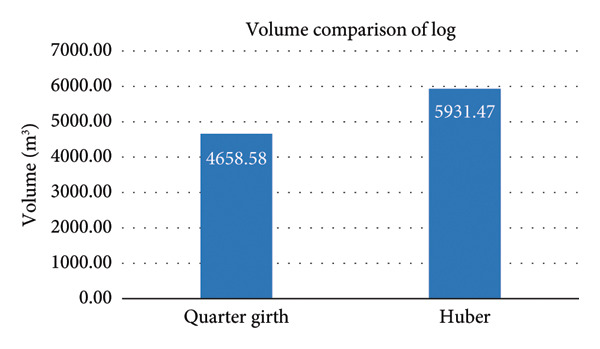
Comparative chart of log volume using the Quarter girth and Huber formula.

The volume of Huber’s formula is estimated to be 27.34% higher than the Quarter girth formula (Figure 4). This indicates a significant difference between the two methods, with Huber’s formula providing an increase in volume compared to the Quarter girth formula.

The Quarter girth formula is a widely used method for estimating log volume based on girth measurements. This formula calculates the volume of plank instead of whole log; also, it assumes the value of pie (*π*) as 4 not 3.14 [19]. Therefore, it underestimates the volume of trees, while Huber’s formula is considered more precise for estimating log volumes, especially for logs that exhibit taper. It is commonly used when accurate measurements of diameter or circumference at various heights on the log are available. The formula considers the changing shape of the log as taper and provides a more realistic representation of the log volume compared to the Quarter girth formula.

### 3.5. Volume Estimated Using Four Different Formulas for Both Standing Trees and Logs

Differences were observed in the total and average tree volume between the formulas (Table 4). The allometric equation for standing timber provided the highest estimate of total volume of sample trees of 7374 m^3^and average tree volume of 44 m^3^. This is then followed by Huber’s formula for logs with a total volume per tree of 5936 m^3^ and average tree volume of 35 m^3^. The form factor formula estimated the total volume of sampled trees at 5869 m^3^ and average tree volume of 35 m^3^. The formula that yielded the lowest volume estimate is the Quarter girth formula with estimated total volume of sample trees at 4659 m^3^and average tree volume of 28 m^3^.

**Table 4:** Total volume of all sample trees, average volume per tree, and variances estimated by the four volume formulas.

Formula	Frequency	Total volume of all sample trees (m^3^)	Average volume per tree (m^3^)	Variance
Form factor–based formula (tree)	168	5868.5	34.9	818.3
Allometric equation (tree)	168	7374.0	43.9	1270.3
Quarter girth (log)	1192	4658.6	27.7	644.1
Huber (log)	1192	5931.5	35.3	1044.1

The ANOVA showed highly significant differences of the average volume per tree (Table 5) between the four formulas with *p*‐value of 0.00004. The Tukey HSD test (Table 6) showed that the Quarter girth and old formula (form factor based) for standing tree is significantly different to allometric equation of [14]. However, the Quarter girth and Huber formula did not show significant differences, while Huber and allometric equation also did not have significant differences.

**Table 5:** ANOVA result.

Source of variation	SS	Df	MS	*F*	*p*‐value	*F* crit
Between groups	22036.77858	3	7345.592859	7.7796068	0.00004	2.618
Within groups	630733.1664	668	944.2113269	—	—	—
Total	652769.9449	671	—	—	—	—

**Table 6:** Tukey’s HSD.

Volume form	*N*	Subset for alpha = 0.05	
		1	2
Quarter girth	168	27.7296	—
Form factor–based formula (ST‐Old)	168	34.9315	—
Huber	168	35.3064	35.3064
Allometric equation (ST‐New)	168	—	43.8927
Sig.	—	0.109	0.052

**Figure 5:**
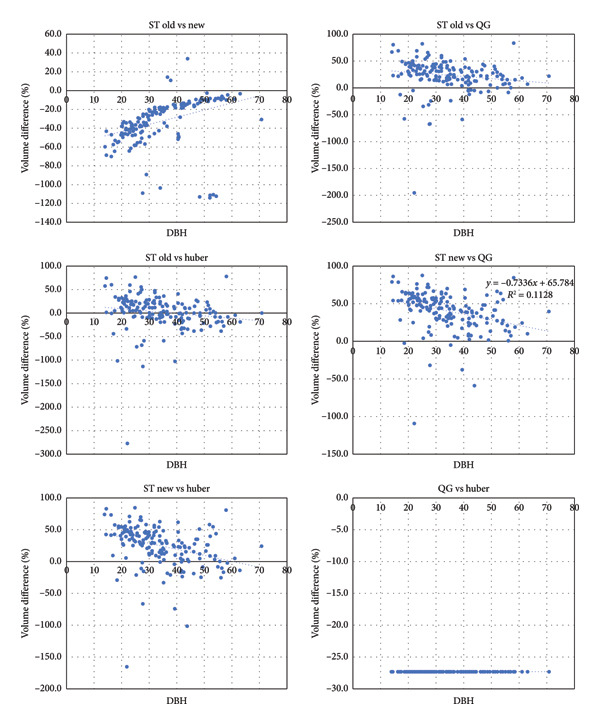
Scatter plots of volume differences (%) between pair of formulas based on DBH on the X axis.

Inspection of the volume differences through scatter plots showed that except for the Quarter girth formula versus Huber’s formula, all other pairwise volume differences showed an inverse relationship with the DBH (Figure 5). This means that the volume difference decreases with increasing DBH with some indication that differences approach zero when tree DBH is more than 30 cm, for example, in Huber’s versus old form factor formula. The flat relationship shown by the difference of the Quarter girth and Huber formula to tree DBH is inherent to its mathematical relationship showing that the Quarter girth estimates about 25% lower cross‐sectional area than that of Huber’s formula.

### 3.6. Difference Between Standing Tree Volume, Felled Tree Volume, and Log Volume

Table 7 presents the total volumes estimated for standing trees, felled trees, and log (*n* = 168 trees). The timber loss rates during conversion from felled tree to logs with the previously used and new formula were 37.06% and 19.86%, respectively. Paired *t*‐test at the 5% level of significance revealed that there was significant timber loss in tree felling stage with the Quarter girth formula (*p*‐value = 7.92 × 10−10). Similarly, there was significant timber loss in tree felling stage with the Huber formula (*p*‐value = 3.20 × 10−7).

**Table 7:** Total volumes of standing trees, felled trees, bucked logs, and timber loss.

Types	Estimated standing tree volume (m^3^)	Actual felled tree volume (m^3^)	Bucked logs volume (m^3^)	Timber loss (%)
	(*a*)	(*b*)	(*c*)	(*e*) = ((*b* − *c*)/*b*) × 100
Form factor–based formula	5868.49	7401.66	4658.58	37.06
Allometric equation	7401.66	7401.66	5931.47	19.86

**Figure 6:**
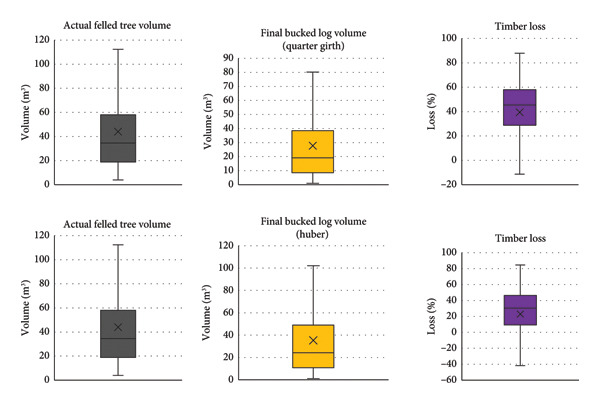
Box plots illustrating the variations in three key aspects during the tree felling stage: actual felled tree volume, final bucked logs, and timber losses by the Quarter girth and Huber formula.

In the form factor–based previously used formula, the volume of the felled tree increased from the standing tree volume by 20.71%. Conversely, upon applying the Quarter girth formula, the volume of the bucked logs decreased by 37.06% compared to the actual felled volume (as depicted in Figure 6), yielding the commercial volume. However, when utilizing the Huber formula, the reduction in bucked log volume was only 19.86% from the actual felled volume (as demonstrated in Figure 6) indicating a comparatively less loss in volume than the Quarter girth formula.

## 4. Discussion

The findings of the study revealed that the implementation of the allometric equation proposed by Sharma and Pukkala [14] resulted in a slight decrease of approximately 0.8% (31.88 m^3^) in the estimated timber volume compared to the volume obtained using the form factor–based formula. However, for firewood, the allometric equation yielded a significantly higher volume of 3554.15 m^3^, whereas the previously used formula resulted in a volume of 1989.10 m^3^, indicating a substantial increase of about 78.6% (1565.05 m^3^). These results align with previous studies conducted by Thakur, 2006; Sharma et al. which concluded that the form factor recommended by the Government of Nepal underestimates the standing volume for pine trees. Furthermore, several other studies have also demonstrated that allometric equations provide superior models for predicting volume or biomass of individual trees [20, 21].

Indeed, it is widely acknowledged in scientific literature that the volume, biomass, and growth of trees typically exhibit a nonlinear pattern. However, it is important to recognize that the specific form of the nonlinear model can vary depending on factors, such as tree species, geographical region, and stand density. This observation has been supported by various studies conducted by researchers [22–24]. These studies highlight the influence of these factors on the shape and characteristics of the nonlinear relationship between tree attributes and volume, biomass, or growth, underscoring the need to consider these factors when developing and applying allometric equations and models in different contexts. In this study, we compared the volume estimation of tree logs using two different formulas: the Huber formula and the Quarter girth formula.

The analysis findings indicate that the Huber formula produced an average log volume, while the Quarter girth formula resulted in a volume averaging 27.34% higher. These findings are consistent with the observations made by Freese in 1974. Notably, a direct comparison between the two formulas revealed a statistically significant difference (*p* < 0.05) in the computed volumes, which aligns with the findings of [25].

The findings from this study indicate that 37.06% and 19.86% of stem volume losses occurred during the felling and bucking operation by the Quarter girth and Huber formula, respectively. The finding corroborates with other studies conducted with similar kinds of formulas. However, more variations in tree harvesting loss rate can be seen in different studies (Table 8).

**Table 8:** Tree harvesting loss rate of previous studies.

Study area	Loss rate (%)	Formula used for standing tree and logs	Authors
Nepal	19.8	Sharma and Pukkala (1990) and Newton	[1]
Nepal	27.0	Sharma and Pukkala (1990) and Newton	[26]
Nepal	21.6	Sharma and Pukkala (1990) and Newton	[25]
Gabon	25	Allometric equation and Smalian’s formula	[27]
Ghana	30	Allometric equation	—
Sarawak Malaysia	46	Allometric equation	—
Australia	47.20	Sharma and Pukkala (1990) and Newton	[1]
Asia	50 (1:1 ratio)	Allometric equation	[28]
The Philippines	60	Allometric equation and Huber	[29]
Brazilian Amazon	66 (1:2 ratio)	Allometric equation and Huber	[30]

The key factors for these losses were associated with stem rot, higher stump, and other logging losses left in the forest after harvesting [1]. Harvesting loss rate during logging varies in different studies depending on its local conditions, often considered 1:1 (i.e., 1 m^3^ of extracted logs result in 1 m^3^ of logging residue) as a thumb rule [31]. Many scholars indicated a wide range of timber loss during selective logging, i.e., one to five times the extracted timber, indicating a recovery rate starting from 20% [28]. The logging loss rate using Huber’s formula estimated in this study is similar to the results of [1] in Nepal. Poudyal et al. [1] conducted a study using Newton formula, adopting a similar methodological framework as in this study. Compared to our study, Poudyal et al. [1] found 19.8% of timber loss. And this result shows that Huber’s formula also has similar amount of timber loss as that in Newton.

Table 8 presents the findings from various studies, revealing that most of them have reported a loss of approximately 50% of the stem volume during timber harvesting. This estimation was based on the utilization of Huber’s, Smalian’s, and Newton’s formulas for log measurement. The observed rates of volume loss are significantly higher in comparison with the findings of our study. Several plausible reasons account for the superior rate of loss. Firstly, transportation losses were considered in the previous studies, but they were excluded in our analysis. Secondly, the recovery rate calculation in the previous studies encompassed multiple species, taking the average recovery rate of timber volume across different species, whereas our investigation focused exclusively on the pine species in Nepal.

## 5. Conclusion

We conducted a comparative analysis of volume estimation for standing trees, utilizing an allometric equation and a previously used formula based on form factor. The results obtained demonstrated statistically significant differences between the two approaches. Specifically, the formula based on form factor exhibited a consistent tendency to underestimate firewood volume in comparison with the allometric equation. These findings underscore the criticality of comprehensively evaluating the limitations and assumptions associated with each method to ensure accurate tree volume estimation. Similarly, the comparative analysis of the Huber formula and the Quarter girth formula for log volume estimation indicates that the Huber formula exhibits significantly less timber loss compared to the Quarter girth formula. ANOVA revealed highly significant differences in the average volume per tree between the four formulas (*p*‐value = 0.00004) for both standing tree and log volume, and further analysis using Tukey’s HSD indicated that the Quarter girth and form factor–based formula for standing trees differed significantly from the allometric equation for standing timber, while other formula combinations did not show significant differences. The Huber formula’s incorporation of Quarter girth contributes to a more accurate estimation of log volume, resulting in reduced timber loss. These findings highlight the implication of selecting appropriate volume estimation methods, such as the Huber formula, in optimizing timber utilization and minimizing timber loss in sustainable forest management. Further research should be conducted to assess the applicability and efficiency of the Huber formula across diverse tree species and varying forest conditions to strengthen its validity and practical implementation.

## Conflicts of Interest

The authors declare no conflicts of interest.

## Funding

No funding was received for this manuscript.

## Data Availability

The data that support the findings of this study are available from the corresponding author upon reasonable request.
